# Supplementary material for: ABCC9-related Intellectual disability Myopathy Syndrome is a KATP channelopathy with loss-of-function mutations in ABCC9
Source: Nat Commun. 2019 Oct 1;10:4457. doi: 10.1038/s41467-019-12428-7 (PMC6773855; doi:10.1038/s41467-019-12428-7)
Supplement: Supplementary file 1 — Supplementary Information [file 41467_2019_12428_MOESM1_ESM.pdf]

Supplementary Information

ABCC9-related Intellectual disability Myopathy Syndrome is a K_{ATP} channelopathy with loss-of-function mutations in *ABCC9*

Smeland et al.

Supplementary Methods

Primers for cDNA analysis of human fibroblast samples

Transcript amplification from patient cDNA spanning *ABCC9* exons 6-9 was performed using a ABCC9_cDNAex6.F forward primer (5'-TTCTGGAATAGTTCAGCGTGTG-3') and a ABCC9_cDNAex9.R reverse primer (5'-ACAGCTGCACCGACCAAT-3').

Mouse model

Behavioral, emotionality and cognitive testing of SUR2-Stop mice

1-hour locomotor activity, exploratory behavior and sensorimotor battery: Baseline locomotor activity was assessed using a transparent polystyrene enclosure (47.6 x 25.4 x 20.6 cm) and computerized photobeam instrumentation as described previously[1,2]. Total ambulatory time and vertical rearings were taken as measures of activity whilst the time spent in the center of the field (33 x 11 cm) and the edges of the field (5.5 cm from walls) were used as indices of emotionality. The next day, mice were subjected to a series of sensorimotor tests selected to assess coordination, balance (ledge, platform, pole and inclined screen tests), strength (screen tests), and initiation of movement (walking initiation), as described previously[1,3]. For the balance tests, mice were placed on a Plexiglass ledge (0.75 cm wide, 30 cm elevation) or a small circular platform (3 cm in diameter, 47 cm elevation) and the time the mice remaining on the ledge/platform was recorded. For the pole test mice were placed head-upwards with forepaws on top of a textured rod (8 mm diameter, 55 cm height) and the time taken for the mouse to turn and descend the pole was recorded. The screen tests were conducted by placing a mouse head-downwards upon a mesh screen (16 squares per 10 cm, elevated 47 cm and angled at 60 or 90o), the time the mouse took to turn and climb to the top of the screen was recorded. The times of two trials for each test were averaged for analysis.

Morris Water Maze Navigation: The day after the sensorimotor battery we employed the MWM using a computerized tracking system (ANY-maze; Stoelting) to assess spatial learning and memory as described previously[1,2]. Cued trials were performed in which a visible platform was variably placed in the water maze with visible cues. Four trials were conducted per day (60 s maximum time) for two consecutive days with the platform being moved to different locations for each new trial using a 30 min inter-trial interval (ITI) and with limited, distal spatial cues being present to limit spatial learning. The time, distance and swimming speed for mice to find the platform was recorded across four blocks of trials (two trials/block). Three days later, place trials were initiated in which the platform was submerged and hidden but remained in a constant location, to determine spatial learning. Mice were required to learn the single location of a submerged platform in relation to spatial cues. Place trial data were recorded from over five blocks of trials (four trials/block), in which each block included the performance level for each of five consecutive days of testing. Finally, a probe trial wherein a mouse was released into the water maze where the platform had been removed was administered ~ 1 h after the final Place Trial (on day 5 of place trial testing). The time spent in the various pool quadrants, including the target quadrant (where the platform had previously been placed), was recorded.

Object recognition test: Elevated Plus Maze: Anxiety-like behavior was measured using the Elevated Plus Maze as previously described[2,4]. The black Plexiglass EPM apparatus consisted of two opposed open arms (without walls) and two opposed closed (walled) arms (36 x 6.1 x 15 cm) which extended in a + shape from a central square platform (5.5 x 5.5 cm). Behavior in the maze was recorded using an automated, computerized recording set up

(Kinder Scientific). Beam break data was recorded and analyzed using MotorMonitor software (Kinder Scientific), distance traveled, time spent in each area and entries into open and closed arms were recorded. Test sessions were performed in a dimly lit room (lighting with 13-watt blacklight bulbs; Ecobulbs, Feit) where each session began by placing a mouse in the center of the maze allowing for free exploration of the maze. Each session lasted 5 min and the mice were tested over 3 consecutive days.

Statistical analyses

Data was analyzed as previously reported[5]. Numerical data were presented as mean ± SEM. ANOVA models were used to test for statistical significance. Repeated-measures ANOVA (rmANOVA) models containing two between-subjects variables (genotype and sex) and once within-subject (repeated measures) variable (e.g. blocks of trials) were typically used to analyze the MWM and EPM data. The Huynh-Fledt adjustment of α levels was used for all within-subject effects containing more than two levels to protect against violations of sphericity/compound symmetry assumptions underlying rmANOVA models. We used one-way ANOVA models to test for between-group differences in the 1 h locomotor activity and sensorimotor tests.

Zebrafish model

Quantitative RT-PC

qPCR was performed using PrimeTime qPCR primer/probe assay. Gapdh forward primer: caggttgtgtccactgactt; reverse primer: gtattgctctcaacgatcactttg; probe: tcatccatctttgacgctggtgct. Abcc9 forward primer: GTGATGAGATTGGAGACGACAG; reverse primer: AAATACCAAGGCGATacgaagg; probe: TGTGCATGTCTCTTGAAGTCGGCA.

Whole-embryo brightfield imaging and measurement of interorbital distance and body length

In vivo phenotypic assessment for whole-embryo imaging were carried out on a Leica M165FC stereomicroscope (Leica Microsystems) with transmitted light. Images were captured with a DFC420 digital microscope camera (Leica Microsystems). Images were applied to measure the distance between the convex tip of the eyes (interorbital distance) in 5 dpf larvae using ImageJ (NIH). To account for variations in size periorbital distance was normalized to overall larval body length. Body length was measured from the tip of the head to the end of the trunk (before the caudal fin).

AFOG staining

Adult zebrafish hearts were dissected and fixed in 4% paraformaldehyde (in Phosphate buffer with 4% sucrose) at 4°C for 4 hours, incubated in PO_4+30% sucrose at 4°C overnight and subjected to embeding in tissue freezing medium (Leica) and sectioning at 10 μm intervals. Acid Fuchsin Orange-G (AFOG) staining was performed as described previously[6]. Image acquisition was conducted using a Leica DM4000 B LED upright automated microscope.

Immunofluorescence

Immunofluorescence was performed in cryosections. PEMTx buffer (80 mM Na-PIPES, 5 mM EGTA, 1 mM MgCl2, pH 7.4; 0.2% TritonX-100) was used for immunohistochemistry. Epitopes were retrieved by heating in citrate buffer (pH 6.0) for 10 minutes at 85°C. Non-specific binding sites were saturated by incubation for at least 1 hour in blocking solution (5% BSA, 0.2% TritonX-100). Primary antibodies used was monoclonal mouse anti-

tropomyosin (1:200; T9283, Sigma). Cy3-conjugated goat anti-mouse secondary antibody (1:500; 115-165-146, Jackson ImmunoR) was used to reveal primary antibody signal. Nuclei were stained with DAPI (4′,6-diamidino-2-phenylindole) and slides were mounted in Vectashield (Vector, Burlingame, CA, USA).

References

1 Wozniak, D. F. *et al.* Apoptotic neurodegeneration induced by ethanol in neonatal mice is associated with profound learning/memory deficits in juveniles followed by progressive functional recovery in adults. *Neurobiol Dis* **17**, 403-414, (2004).

2 Dougherty, J. D. *et al.* The disruption of Celf6, a gene identified by translational profiling of serotonergic neurons, results in autism-related behaviors. *J Neurosci* **33**, 2732-2753, (2013).

3 Grady, R. M., Wozniak, D. F., Ohlemiller, K. K. & Sanes, J. R. Cerebellar synaptic defects and abnormal motor behavior in mice lacking alpha- and beta-dystrobrevin. *J Neurosci* **26**, 2841-2851, (2006).

4 Schaefer, M. L. *et al.* Altered stress-induced anxiety in adenylyl cyclase type VIII-deficient mice. *J Neurosci* **20**, 4809-4820 (2000).

5 Stein, L. R. *et al.* Expression of Nampt in hippocampal and cortical excitatory neurons is critical for cognitive function. *J Neurosci* **34**, 5800-5815, doi:10.1523/JNEUROSCI.4730-13.2014 (2014).

6 Poss, K. D., Wilson, L. G. & Keating, M. T. Heart regeneration in zebrafish. *Science* **298**, 2188-2190, (2002).

Supplementary Figure 1

Regions of homozygosity in the *ABCC9* gene regions of patients 1-3, 1-4, 2-1 and 2-2.

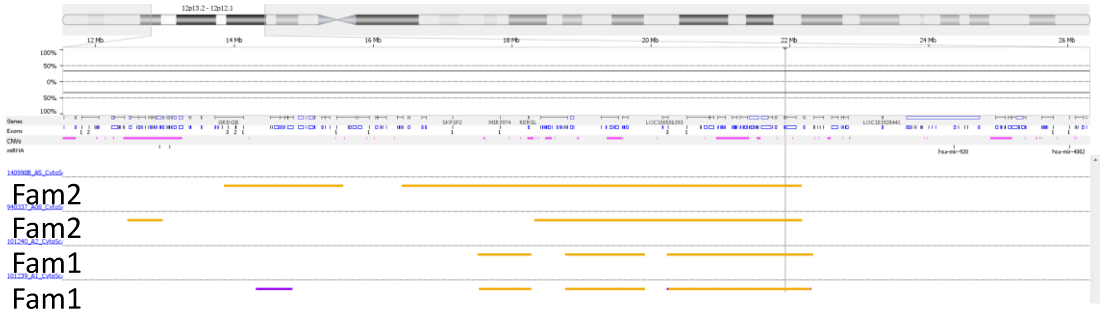

Supplementary Figure 2

Shared homozygous variant in patients 1-2 and 2-1.

Cohort details for position: 12:22,063,090

Variants

Patients	Gene	Position	Type	Transcript	cDNA	Location	Exon	Effect	Protein
2 / 2	ABCC9	12:22,063,090	snp	NM_020297.2	c.1320+1G>A	intronic	8		

Patients

Export

Patient Accession No.	Group	Family Id	Comments	Ref	Patient	Read Depth	Call Quality	Genotype Quality	Mapping Quality	Filter status	Info	Actions
P_NGS150131_CLS	Case			C	T T	36	2,131.2	99		PASS		
P_NGS170003_CLS	Case			C	T T	35	2,131.2	99		PASS		

Supplementary Figure 3

Shared Homo-, Hemi- and VarX-VarY variants.

Gene	Cases	Controls	Difference	Position	Ref	Alt	Read Depth	Type	Transcript	cDNA	Location
GFI1			2	1:92,944,314	.	AGAG···G		insertion	NM_005263.3	c.925-5_925-4insCTCTCTCTCT	intronic
NT5DC2			1	3:52,567,792-52,567,799	CCAG···G	C		deletion	NM_001134231.1	c.-46_-40delCGGGCTG	UTR5
NT5DC2			1	3:52,567,792-52,567,799	CCAG···G	CCCG···G		substitution	NM_001134231.1	c.-46_-39delCGGGCTGGinsCGGGCTGCGGGCGGCGGGCGGG	UTR5
HLA-DRB1			2	6:32,552,144-32,552,145	AC	A		deletion	NM_002124.3	c.111delG	exonic
ADAM12			1	10:128,019,025	C	A		snp	NM_003474.5	c.142G>T	exonic
CCDC15			1	11:124,875,006	G	A		snp	NM_025004.2	c.2309G>A	exonic
PIK3C2G			1	12:18,719,887	C	T		snp	NM_001288772.1	c.3907C>T	exonic
ABCC9			2	12:22,063,090	C	T		snp	NM_020297.2	c.1320+1G>A	intronic

Supplementary Figure 4

Compound heterozygous gene candidate analysis.

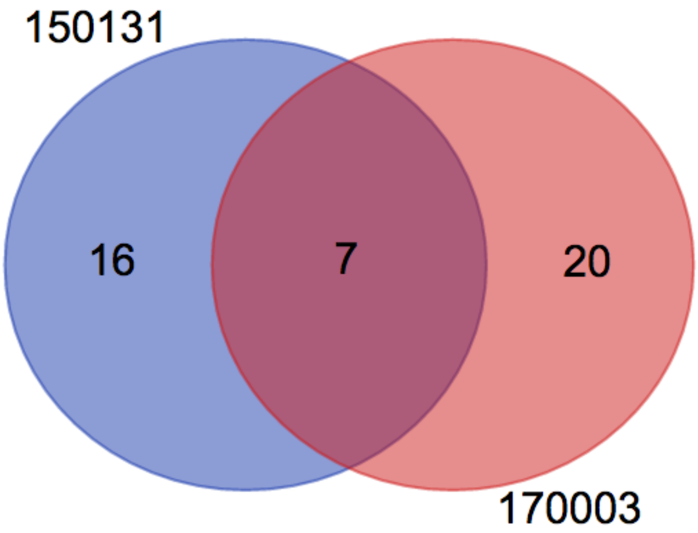

Names	total	elements
150131 170003	7	ZNF717 CTDSP2 CDC27 MUC3A MUC16 MUC19 USH2A
150131	16	ZNF486 TTN SOX2 DYSF CD48 NBPF1 HMCN1 ARL13B ZNF780A TMEM200C LRP1 SERPINA1 TECTA ERVW-1 RND2 NT5DC2
170003	20	ESPL1 MUC17 AHNAK2 ZNF626 ZNF577 LAMA5 OBSCN HLA-DRB1 ZNF736 OTOA ZNF469 ART5 RABGAP1L COL11A2 PRRC2A PASK NLRP2 CRIPAK MUC21 CTBP2

Supplementary Figure 5

Pinacidil sensitivity is unchanged in recombinant mixed SUR2A-WT/SUR2AΔ8 channels.

(A) Example inside-out patch clamp recordings from Cosm6 cells transfected with Kir6.2 and SUR2A-WT or a 1:1 mix of SUR2A-WT and SUR2AΔ8. Patches were voltage clamped at -50 mV and administered ATP (in the presence of 0.5 mM free Mg^{2+}) and pinacidil as indicated. (B) Summary of the extent of pinacidil activation. Currents in the presence of pinacidil were normalized to currents in the absence of nucleotides. Source data are provided as a Source Data file.

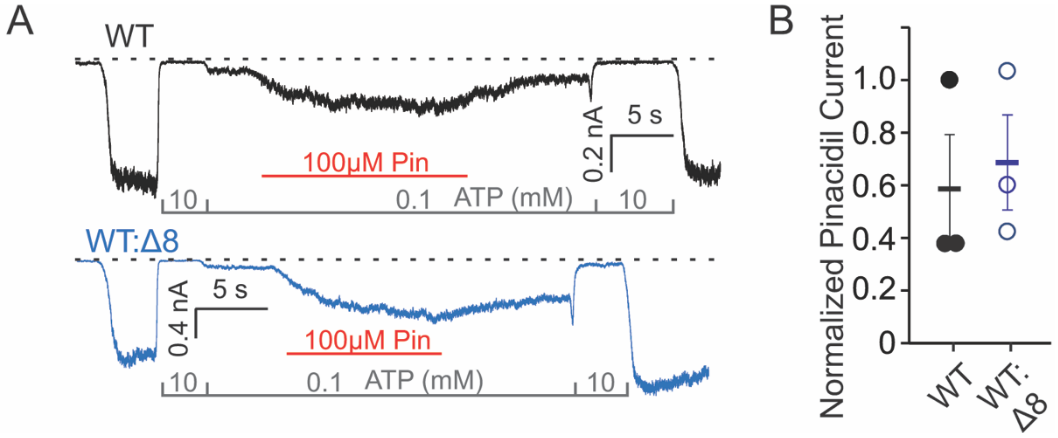

Supplementary Figure 6

Loss of K_{ATP} channel function in aortic smooth muscle, and increased blood pressures in SUR2-STOP mice.
(A) Example whole cell patch clamp recordings from acutely isolated aortic smooth muscle cells. Cells were voltage clamped at -70 mV and KATP channels were activated in high-K^+ extracellular solution by administration of pinacidil (100 µM) as indicated. (B) I_{KATP} was determined as the glibenclamide-sensitive, pinacidil-activated current. * denotes $p < 0.05$ according to students T-test. (C) Example recordings of arterial blood pressures in WT and SUR2-STOP mice. (D) Summary of mean arterial pressure (MAP). * denotes $p < 0.05$ according to students T-test. Source data are provided as a Source Data file.

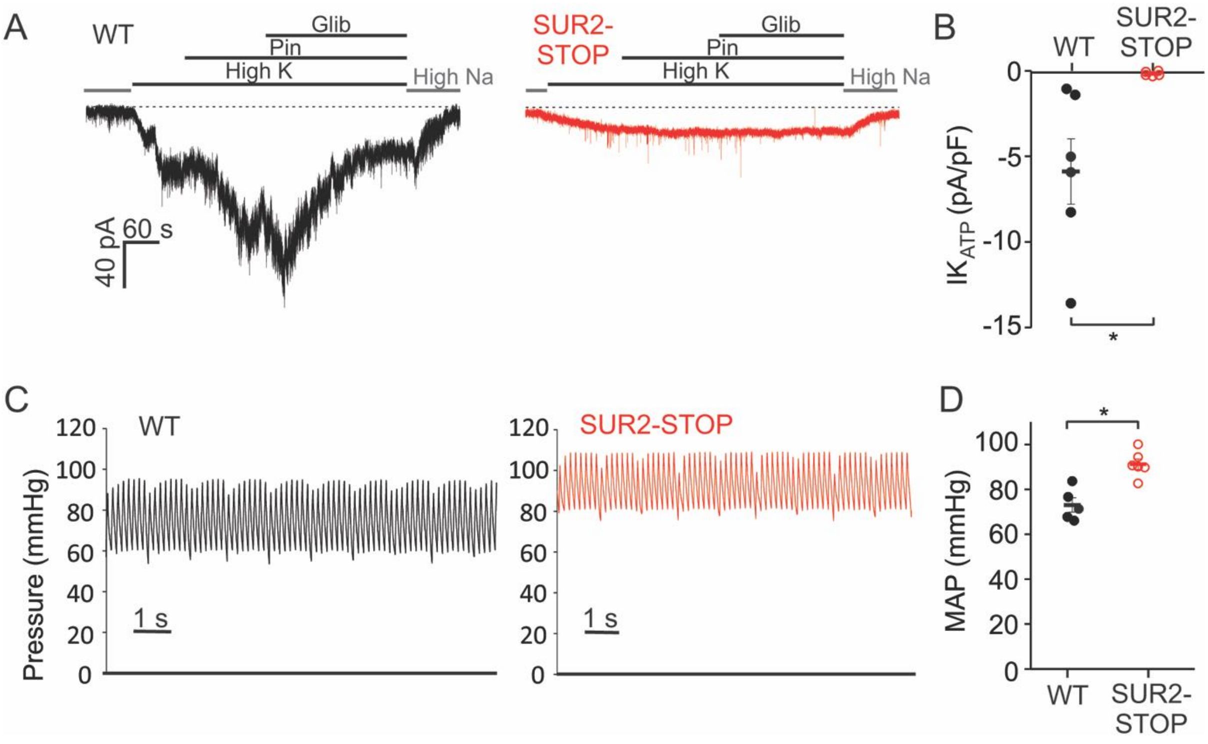

Supplementary Figure 7

SUR2-STOP mutation induces systolic dysfunction in zebrafish larvae.
(A) Quantification of stroke volume and heart beat applying individual characteristic confocal sections from a time series of the embryonic cardiac cycle at 5 dpf. For all graphs, significance was determined by two-tailed unpaired Student's t test or Mann–Whitney two-tailed U test. Asterisks indicate statistical significance (* p≤0.05; ** p≤0.01; *** p≤0.001; **** p≤0.0001). The black horizontal bar indicates the mean value for each condition. Data shown as mean ± SEM. Sample size, WT, n=20; SUR2-STOP, n=20 in. All embryos analysed originated from group matings of adult zebrafish. Source data are provided as a Source Data file.

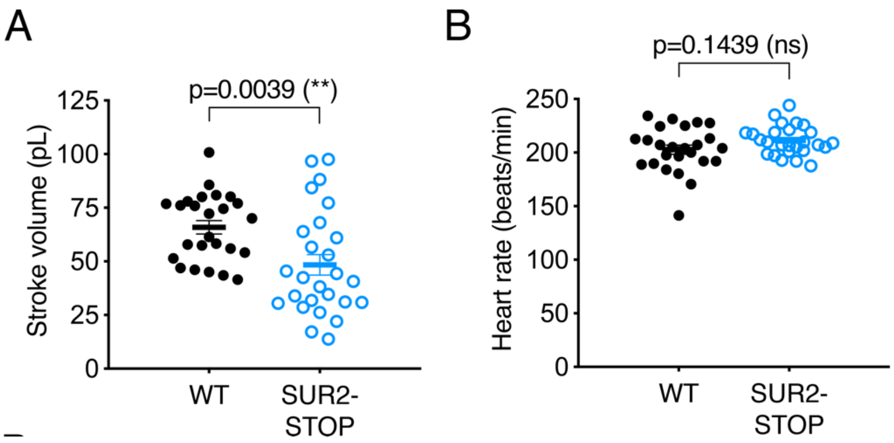

Supplementary Figure 8

Assessment of ventricular contractility via high-speed video microscopy in zebrafish larvae.
Representative images illustrating the morphology of 5 dpf wild-type (A) and SUR2-STOP (B) mutant hearts at diastole and systole as seen from a dorsal view. The ventricular area of the heart is highlighted, with the ventricular diameter D_d and D_s indicated by a grey arrow. Fractional shortening (FS), a measure of ventricular contractility, was calculated from ventricular diameters at diastole and systole (D_d and D_s).

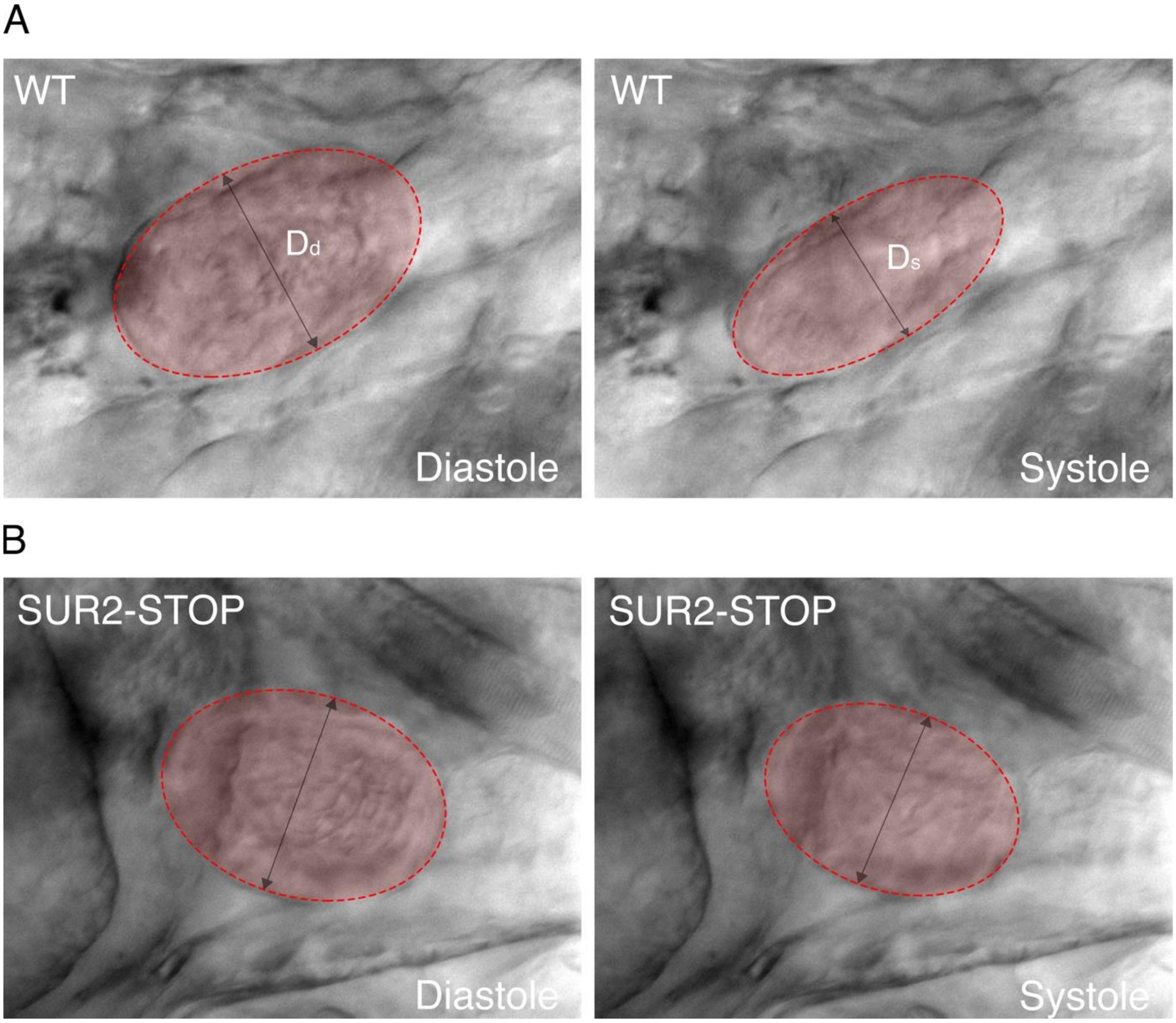

Supplementary Figure 9

Zebrafish larvae heterozygous for SUR2-STOP mutation lack cardiac abnormalities. (A)-(G) Quantification of cardiac function in 5 dpf zebrafish larvae heterozygous for SUR2-STOP mutation using individual characteristic confocal sections from a time series of the embryonic cardiac cycle. WT controls are the same as in Fig. 7B-E and Fig. S5A. For all graphs, significance was determined by two-tailed unpaired Student's t test or Mann–Whitney two-tailed U test. The black horizontal bar indicates the mean value for each condition. Data shown as mean ± SEM. Sample size, WT, n=25; WT/SUR2-STOP, n=21 in **A-G**. All embryos analysed originated from group matings of adult zebrafish. Source data are provided as a Source Data file.

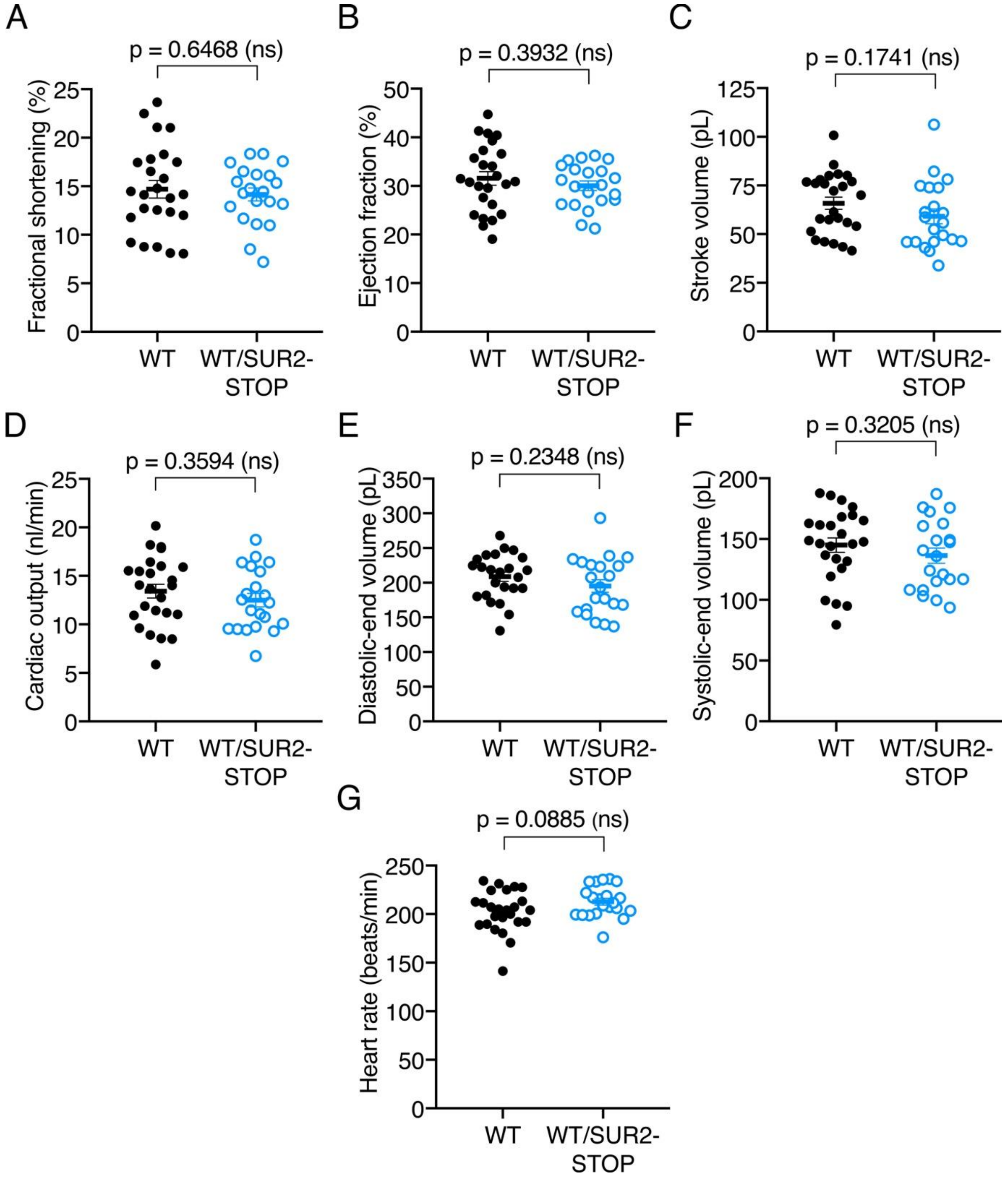

Supplementary Figure 10

SUR2-STOP mutation induces enlarged heart size in adult zebrafish.
Heart histology of WT siblings (A-D) adult SUR2-STOP mutants (A'-D') after H&E staining. Depiction of four WT and four SUR2-STOP hearts. For assessment of ventricular chamber size, tissue sections showing the largest ventricular area were selected. Sample size, WT, n=6, SUR2-STOP, n=6. Scale bar, 500 μm.

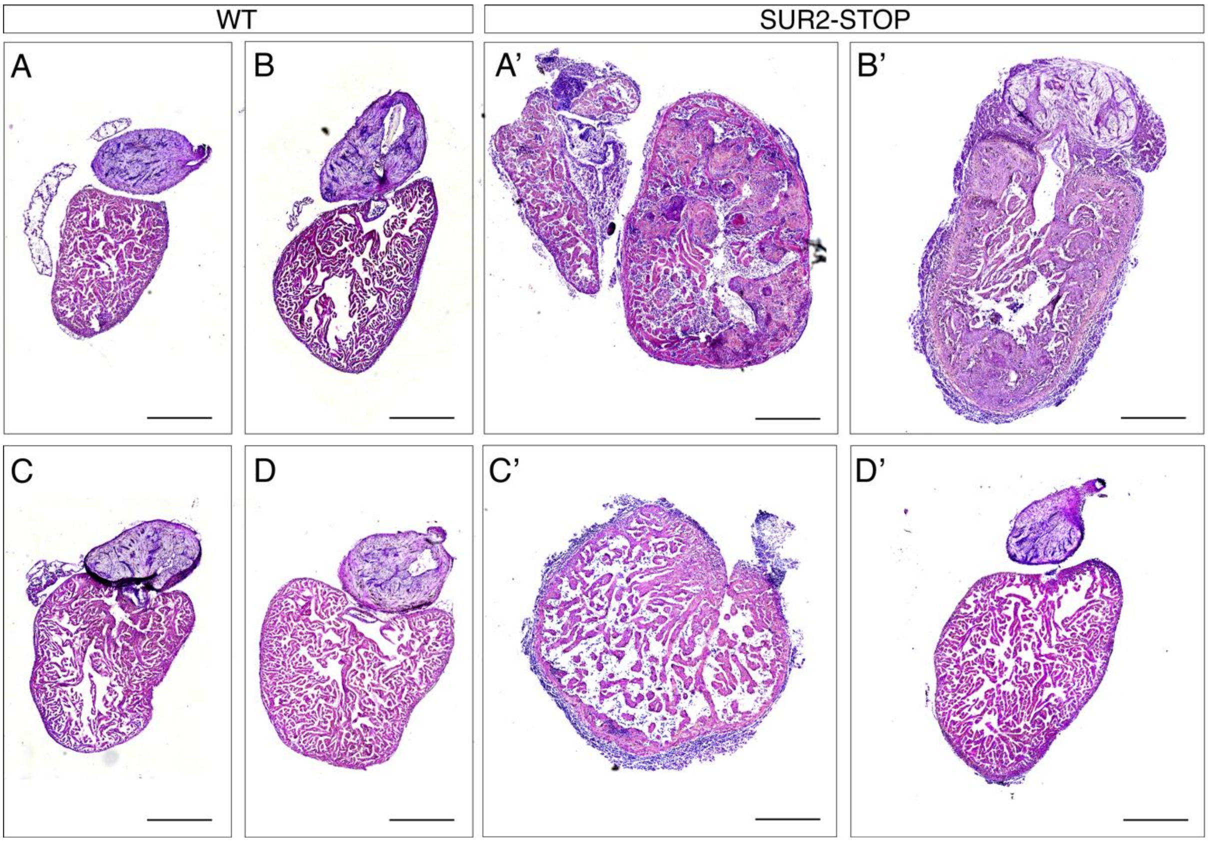

Supplementary Figure 11

No fibrosis in hearts of adult heart of SUR2-STOP.
Heart histology of adult Wildtype (A-F) and SUR2-STOP mutants (A'-F') after Acid Fuchsin Orange G (AFOG) staining, which labels myocardium orange, collagen blue and fibrin red. Depiction of 6 WT and 6 SUR2-STOP hearts. Notably, we selected images for presence of atrium and ventricle to show staining of both chambers; not for the largest ventricular area. (G-H) Positive control showing two adult zebrafish hearts 7 days after cryoinjury presenting with red staining to show fibrosis around the apex. Sample size, WT, n=6, SUR2-STOP, n=6. Scale bar, 500 μm.

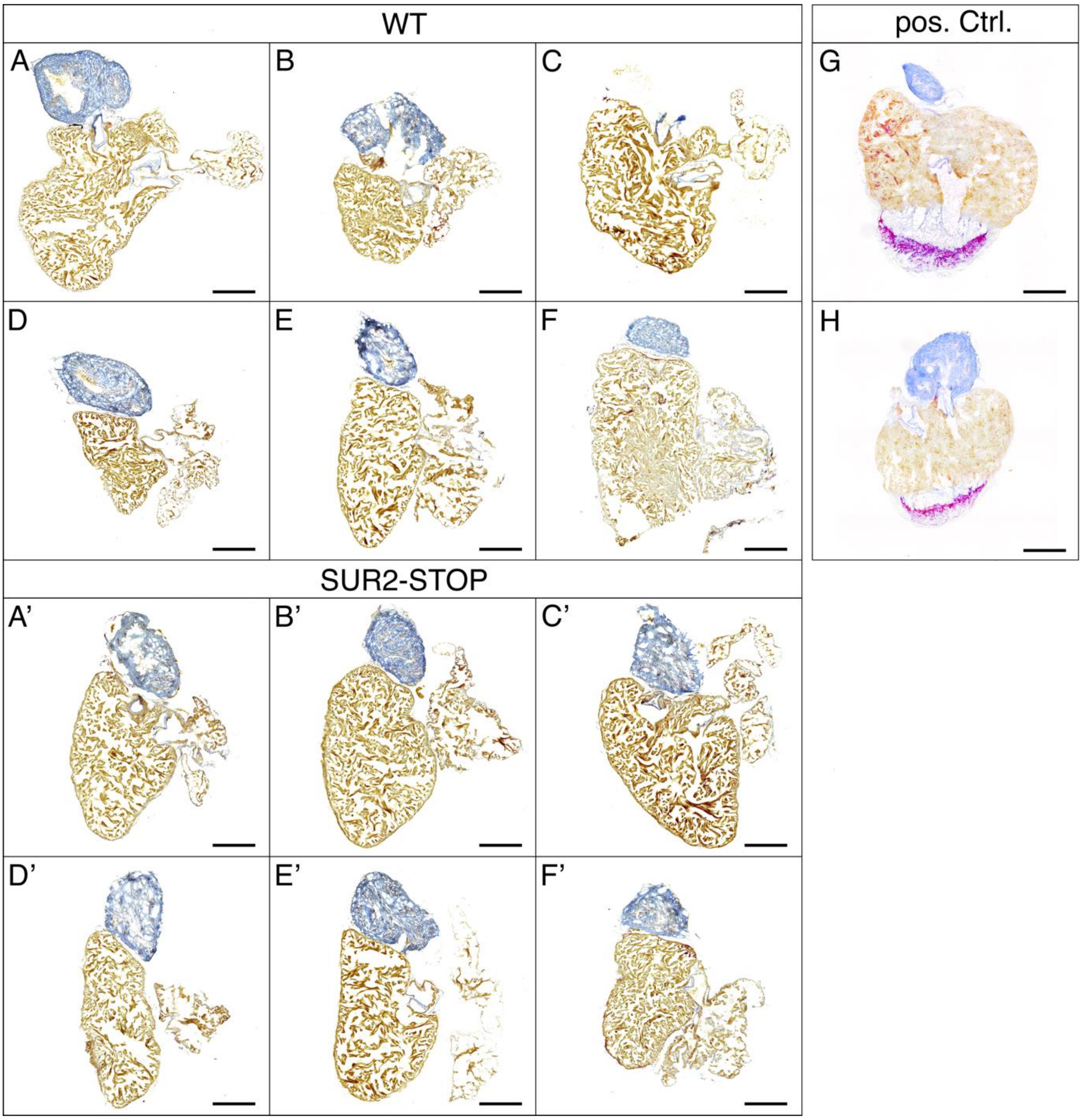

Supplementary Figure 12

SUR2-STOP fish show no abnormalities in myofiber structure.
Immunohistochemistry on sagittal sections of adult hearts of WT (A) and SUR2-STOP fish (A') with antibody against to tropomyosin (red); nuclei are stained with DAPI (blue). To assess structure of myofibers four different areas were assessed: center of ventricle, apex, ventricular border and atrium. Areas are indicated in boxes. (B-E) and (B′-E′) Higher magnification views of the boxed areas in A and A'. Arrowheads indicate stained tropomyosin. Sample size, WT, n=6, SUR2-STOP, n=6. Scale bars, 500 μm in **A** and **A'**; 100 μm in **B-E and B'-E'.**

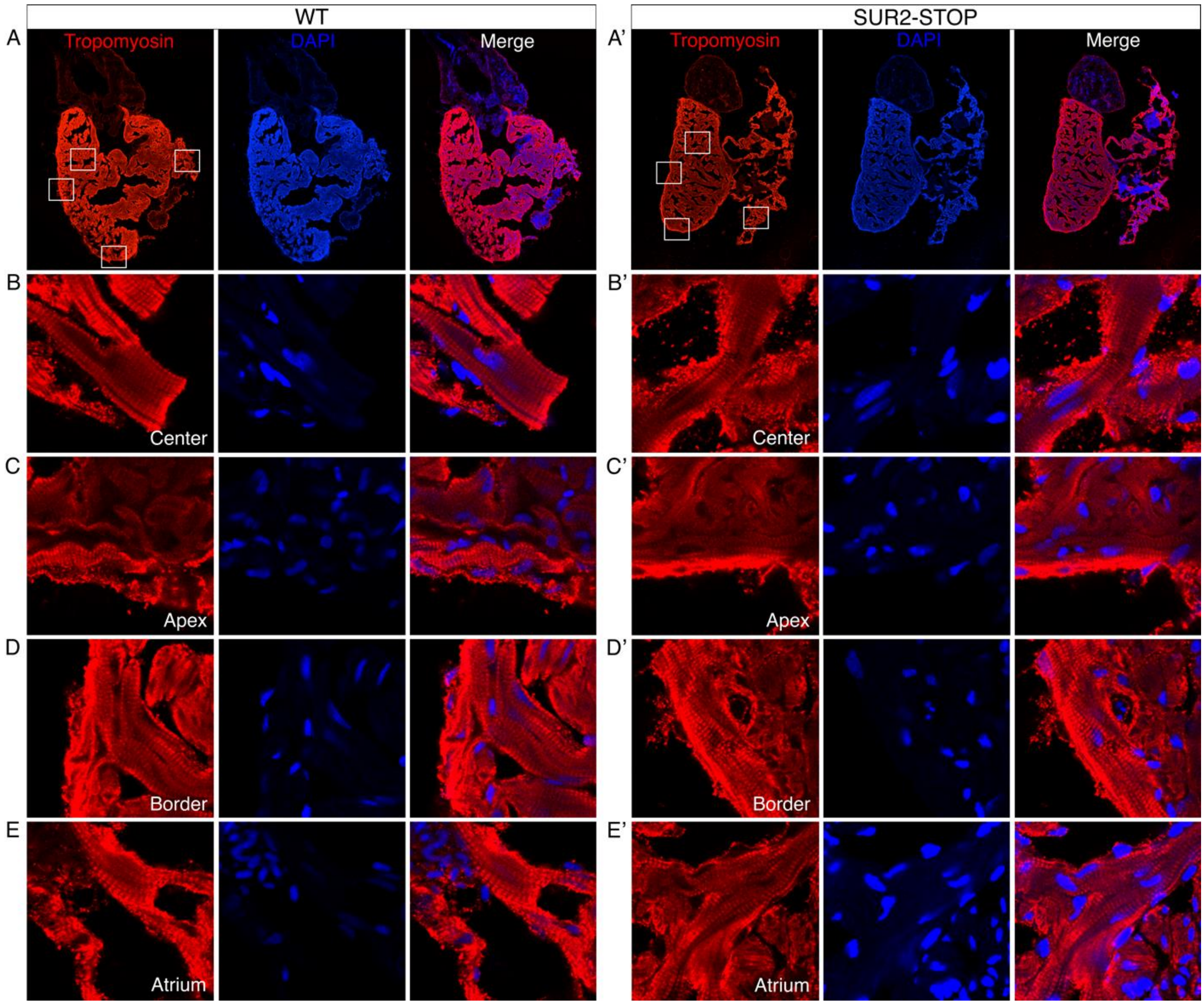

Supplementary Table 1

Whole genome sequencing quality statistics for patient 1-2 and 2-1.

	**Coverage statistics**	
	NGS150131_dedup	NGS170003_dedup
Total number of reads	836441988	713843771
Percentage reads mapped	98.69%	99.01%
GENOME_TERRITORY	2858674662	2858674662
MEAN_COVERAGE	3.238.531	2.783.482
SD_COVERAGE	1.130.788	1.057.191
MEDIAN_COVERAGE	32	28
MAD_COVERAGE	6	6
PCT_EXC_MAPQ	0.052223	0.049995
PCT_EXC_DUPE	0.137133	0.133782
PCT_EXC_UNPAIRED	0.004893	0.005329
PCT_EXC_BASEQ	0.003871	0.003954
PCT_EXC_OVERLAP	0.021847	0.024628
PCT_EXC_CAPPED	0.01043	0.01012
PCT_EXC_TOTAL	0.230398	0.227809
PCT_5X	0.986623	0.985449
PCT_10X	0.980767	0.974341
PCT_15X	0.960780	0.932274
PCT_20X	0.913031	0.825773
PCT_25X	0.809961	0.641745
PCT_30X	0.628778	0.419392
PCT_40X	0.215735	0.093175
PCT_50X	0.032871	0.010065
PCT_60X	0.004915	0.002882
PCT_70X	0.002727	0.002092
PCT_80X	0.002146	0.001667
PCT_90X	0.001759	0.001379
PCT_100X	0.001484	0.001154

Supplementary Table 2

Regions of ROH shared amongst patients 1-3, 1-4, 2-1 and 2-2.

Region	Region Length	Region Length in Mb	Cytoband	Event	Genes	miRNAs	Frequency %	#Gene Symbols
chr1:189,555,782-189,758,684	202902	0,202902	q31.1	LOH	0	0	100.0	0
chr2:39,154,670-39,442,510	287840	0,28784	p22.1	LOH	4	0	100.0	4
chr3:41,680,774-42,094,063	413289	0,413289	p22.1	LOH	1	0	100.0	1
chr3:50,524,533-51,875,962	1351429	1,351429	p21.31 - p21.2	LOH	16	0	100.0	16
chr3:96,548,071-97,185,117	637046	0,637046	q11.2	LOH	1	0	100.0	1
chr4:33,301,988-34,236,688	934700	0,9347	p15.1	LOH	1	0	100.0	1
chr4:81,668,252-81,799,259	131007	0,131007	q21.21	LOH	1	0	100.0	1
chr4:148,012,340-148,313,086	300746	0,300746	q31.22	LOH	0	1	100.0	0
chr6:100,950,001-101,848,805	898804	0,898804	q16.3	LOH	2	0	100.0	2
chr7:98,830,528-99,229,189	398661	0,398661	q22.1	LOH	16	0	100.0	16
chr7:118,618,585-119,120,388	501803	0,501803	q31.31	LOH	0	0	100.0	0
chr7:124,365,856-124,897,654	531798	0,531798	q31.33	LOH	6	0	100.0	6
chr8:48,455,013-49,036,395	581382	0,581382	q11.21	LOH	5	0	100.0	5
chr8:49,195,458-49,343,312	147854	0,147854	q11.21	LOH	0	0	100.0	0
chr10:74,671,741-75,300,211	628470	0,62847	q22.1 - q22.2	LOH	15	0	100.0	15
chr10:88,140,718-88,363,018	222300	0,2223	q23.2	LOH	1	0	100.0	1
chr11:47,940,999-50,513,955	2572956	2,572956	p11.2 - p11.12	LOH	17	1	100.0	17
chr12:18,766,066-19,923,554	1157488	1,157488	p12.3	LOH	5	0	100.0	5
<u>chr12:20,258,863-22,176,010</u>	<u>1917147</u>	<u>1,917147</u>	<u>p12.2 - p12.1</u>	<u>LOH</u>	<u>15</u>	<u>0</u>	<u>100.0</u>	<u>15</u>
chr12:44,165,488-44,863,441	697953	0,697953	q12	LOH	3	0	100.0	3
chr14:61,711,805-62,225,990	514185	0,514185	q23.1 - q23.2	LOH	8	0	100.0	8
chr18:30,383,306-30,885,552	502246	0,502246	q12.1	LOH	1	0	100.0	1

Supplementary Table 3

**Supplementary Table 3. ANOVA effects for multiple trial inverted screen test.**	
**Overall effects**	**F Statistics**
Genotype	*F*(1,16)=15.84, *p*=0.001
Session	*F*(1,16)=8.46, *p*=0.010
Genotype x Session	*F*(1,16)=7.13, *p*=0.017
Trial	*F*(2,32)=2.30, ns (*p*=0.12)
Genotype x Trial	*F*(2,32)=8.93, *p*=0.0008
Session x Trial	*F*(2,32)=0.44, ns (*p*=0.61)
Genotype x Trial x Session	*F*(2,32)=2.19, ns (*p*=0.14)
**Pair-wise comparisons**	
Session 1, Trial 1	*F*(1,16)=1.14, ns (*p*=0.30)
Session 1, Trial 2	*F*(1,16)=3.99, ns (*p*=0.063)
Session 1, Trial 3	*F*(1,16)=12.46, *p*=0.003
Session 2, Trial 1	*F*(1,16)=7.83, *p*=0.013
Session 2, Trial 2	*F*(1,16)=12.79, *p*=0.003
Session 2, Trial 3	*F*(1,16)=40.61, *p*<0.00005
