# Supplementary material for: ABCC9-related Intellectual disability Myopathy Syndrome is a KATP channelopathy with loss-of-function mutations in ABCC9
Source: Nat Commun. 2019 Oct 1;10:4457. doi: 10.1038/s41467-019-12428-7 (PMC6773855; doi:10.1038/s41467-019-12428-7)
Supplement: Supplementary file 3 — Reporting Summary [file 41467_2019_12428_MOESM3_ESM.pdf]

nature research

Corresponding author(s): Dr. Gijs van Haaften
Dr. Marie F. Smeland

Last updated by author(s): 22/07/2019

Reporting Summary

Nature Research wishes to improve the reproducibility of the work that we publish. This form provides structure for consistency and transparency in reporting. For further information on Nature Research policies, see Authors & Referees and the Editorial Policy Checklist.

Statistics

For all statistical analyses, confirm that the following items are present in the figure legend, table legend, main text, or Methods section.

n/a	Confirmed	
☐	☒	The exact sample size (n) for each experimental group/condition, given as a discrete number and unit of measurement
☐	☒	A statement on whether measurements were taken from distinct samples or whether the same sample was measured repeatedly
☐	☒	The statistical test(s) used AND whether they are one- or two-sided<br>*Only common tests should be described solely by name; describe more complex techniques in the Methods section.*
☐	☒	A description of all covariates tested
☐	☒	A description of any assumptions or corrections, such as tests of normality and adjustment for multiple comparisons
☐	☒	A full description of the statistical parameters including central tendency (e.g. means) or other basic estimates (e.g. regression coefficient) AND variation (e.g. standard deviation) or associated estimates of uncertainty (e.g. confidence intervals)
☐	☒	For null hypothesis testing, the test statistic (e.g. F, t, r) with confidence intervals, effect sizes, degrees of freedom and P value noted<br>*Give P values as exact values whenever suitable.*
☒	☐	For Bayesian analysis, information on the choice of priors and Markov chain Monte Carlo settings
☒	☐	For hierarchical and complex designs, identification of the appropriate level for tests and full reporting of outcomes
☒	☐	Estimates of effect sizes (e.g. Cohen's d, Pearson's r), indicating how they were calculated

Our web collection on statistics for biologists contains articles on many of the points above.

Software and code

Policy information about availability of computer code

Data collection	Clampex (Molecular Devices), Zebralab locomotion tracking software (ViewPoint Behavior Technology), Hokawo 2.1 imaging software (Hamamatsu Photonics), LAS X (Leica Microsystems)
Data analysis	MiSeq Reporter Enrichment Workflow (v.2.4.60.8) including a Burrows-Wheeler Aligner (v.0.6.1-r104-tpx) and a Genome Analysis Toolkit (v.1.6-22-g3ec78bd), Cartagenia Bench Lab NGS (v.4), Microsoft Excel (Microsoft), Clampfit (Molecular Devices), ImageJ 2.0.0-rc-43 (NIH), Graph Pad Prism V7 (GraphPad Software)

For manuscripts utilizing custom algorithms or software that are central to the research but not yet described in published literature, software must be made available to editors/reviewers. We strongly encourage code deposition in a community repository (e.g. GitHub). See the Nature Research guidelines for submitting code & software for further information.

Data

Policy information about availability of data

All manuscripts must include a data availability statement. This statement should provide the following information, where applicable:

- Accession codes, unique identifiers, or web links for publicly available datasets
- A list of figures that have associated raw data
- A description of any restrictions on data availability

The authors declare that all data supporting the findings of this study are available within the paper and its supplementary information. Source data for figures 3, 4, 5, 6, and 7, and Supplementary Figures 5, 6, 7, and 9 are provided with the paper.

Field-specific reporting

Please select the one below that is the best fit for your research. If you are not sure, read the appropriate sections before making your selection.

☒ Life sciences ☐ Behavioural & social sciences ☐ Ecological, evolutionary & environmental sciences

For a reference copy of the document with all sections, see nature.com/documents/nr-reporting-summary-flat.pdf

Life sciences study design

All studies must disclose on these points even when the disclosure is negative.

Sample size

The patients in the article were included from diagnostic testing, and so there was no formal pre-planned study.
No sample-size calculations were performed. The sample size was chosen based on considerations of sample sizes used in similar published analyses, and the need to minimise the use of animal subjects where possible (mouse studies and adult zebrafish studies).

Data exclusions

No data was excluded from the analysis.

Replication

Experiments were repeated so that our data are based on at least two to three independent experiments. All experimental findings were reliably reproducible.
Rubidium flux and electrophysiology experiments were conducted on 3 or more different days, using independent transfections (for RB flux) or different mice (for ephyz).

Randomization

We did not use randomization to assign animals to experimental groups.
All available genotypically confirmed mice were used.
Zebrafish larvae were offspring from littermates. Zebrafish adults analyzed were litter mates. Adult fish were sex-matched, identification of sex in larvae was not possible.

Blinding

For behavioural studies of mice investigators were blinded to genotype during data collection and analysis.
For zebrafish studies Investigator was blinded to zebrafish genotype during data analysis.

Reporting for specific materials, systems and methods

We require information from authors about some types of materials, experimental systems and methods used in many studies. Here, indicate whether each material, system or method listed is relevant to your study. If you are not sure if a list item applies to your research, read the appropriate section before selecting a response.

Materials & experimental systems

n/a	Involved in the study
☐	☒ Antibodies
☐	☒ Eukaryotic cell lines
☐	☐ Palaeontology
☐	☒ Animals and other organisms
☐	☒ Human research participants
☐	☒ Clinical data

Methods

n/a	Involved in the study
☐	☐ ChIP-seq
☐	☐ Flow cytometry
☐	☐ MRI-based neuroimaging

Antibodies

Antibodies used

Anti-FLAG M2-HRP conjugate (mouse monoclonal), Millipore Sigma, cat # , Goat anti-mouse IgG antibody-HRP conjugate, Santa Cruz Biotechnology cat # sc-2005, Anti-actin, clone c4 (mouse monoclonal), EMD Millipore, cat # MAB1501, monoclonal mouse anti-tropomyosin (1:200; T9283, Sigma), Cy3-conjugated goat anti-mouse secondary antibody (1:500; 115-165-146, Jackson ImmunoR)

Validation

Antibodies were used according to suppliers instructions. Each antibody has been widely used as documented by supplier (Monoclonal ANTI-FLAG® M2 antibody produced in mouse, https://www.sigmaaldrich.com/catalog/product/sigma/f3165?lang=en&region=US&gclid=CjwKCAiA7vTiBRAqEiwA4NTO6_6PfzbkpfCnCzyXbm2Mr8GkB9mZ_J8WY12RCN6kkqURnu89SeGeDRoCehsQAvD_BwE, 2046 references listed; Goat anti-mouse IgG-HRP, https://www.scbt.com/scbt/product/goat-anti-mouse-igg-hrp, 942 references listed; Anti-Actin Antibody, clone C4, http://www.emdmillipore.com/US/en/product/Anti-Actin-Antibody-clone-C4,MM_NF-MAB1501#anchor_REF, 553 references listed, Cy3-conjugated goat anti-mouse antibody, https://www.jacksonimmuno.com/catalog/products/115-165-146, mouse anti-tropomyosin antibody, https://www.sigmaaldrich.com/catalog/product/sigma/t9283?lang=en&region=NL)

Eukaryotic cell lines

Policy information about cell lines

Cell line source(s)	Cosm6 cells, Chlorocebus aethiops (Green monkey) kidney cells
Authentication	None
Mycoplasma contamination	Cell lines were not tested for mycoplasma contamination
Commonly misidentified lines (See ICLAC register)	N/A

Palaeontology

Specimen provenance	N/A
Specimen deposition	N/A
Dating methods	N/A

☐ Tick this box to confirm that the raw and calibrated dates are available in the paper or in Supplementary Information.

Animals and other organisms

Policy information about studies involving animals; ARRIVE guidelines recommended for reporting animal research

Laboratory animals	SUR2-STOP mice (and WT littermates) were generated by CRISPR-Cas9 genome engineering of B6CBA F1/J embryos. Mice positively genotyped for the mutation were backcrossed against C57BL6/J for 2 generations. Male and female mice were used between the ages of 3 and 6 months.<br>SUR2-STOP zebrafish (and WT littermates) were generated by CRISPR-Cas9 genome engineering of Danio rerio wild-type strain Tübingen longfin. Males and female zebrafish were used between the ages of 6 and 12 months. Zebrafish larvae were used up to 5 days post fertilization.
Wild animals	This study did not involve wild animals.
Field-collected samples	This study did not involve field-collected samples.
Ethics oversight	All mouse studies were performed in compliance with the standards for the care and use of animal subjects defined in the NIH Guide for the Care and Use of Laboratory Animals and were reviewed and approved by the Washington University Institutional Animal Care and Use Committee.<br>All zebrafish studies were conducted under the guidelines of the animal welfare committee of the Royal Netherlands Academy of Arts and Sciences (KNAW).

Note that full information on the approval of the study protocol must also be provided in the manuscript.

Human research participants

Policy information about studies involving human research participants

Population characteristics	The patients in the article were included from diagnostic testing, and so there was no formal pre-planned study.
Recruitment	See above.
Ethics oversight	See above.

Note that full information on the approval of the study protocol must also be provided in the manuscript.

Clinical data

Policy information about clinical studies

All manuscripts should comply with the ICMJE guidelines for publication of clinical research and a completed CONSORT checklist must be included with all submissions.

Clinical trial registration	See above, there was no clinical trial.
Study protocol	N/A
Data collection	N/A

Outcomes | N/A

ChIP-seq

Data deposition

- [ ] Confirm that both raw and final processed data have been deposited in a public database such as GEO.
- [ ] Confirm that you have deposited or provided access to graph files (e.g. BED files) for the called peaks.

Data access links<br>*May remain private before publication.*	N/A
Files in database submission	N/A
Genome browser session<br>(e.g. UCSC)	N/A

Methodology

Replicates	N/A
Sequencing depth	N/A
Antibodies	N/A
Peak calling parameters	N/A
Data quality	N/A
Software	N/A

Flow Cytometry

Plots

Confirm that:

- [ ] The axis labels state the marker and fluorochrome used (e.g. CD4-FITC).
- [ ] The axis scales are clearly visible. Include numbers along axes only for bottom left plot of group (a 'group' is an analysis of identical markers).
- [ ] All plots are contour plots with outliers or pseudocolor plots.
- [ ] A numerical value for number of cells or percentage (with statistics) is provided.

Methodology

Sample preparation	N/A
Instrument	N/A
Software	N/A
Cell population abundance	N/A
Gating strategy	N/A

- [ ] Tick this box to confirm that a figure exemplifying the gating strategy is provided in the Supplementary Information.

Magnetic resonance imaging

Experimental design

Design type	Diagnostic cerebral MRI was performed in all the subjects.
Design specifications	N/A
Behavioral performance measures	N/A

Acquisition

Imaging type(s)	N/A
Field strength	N/A
Sequence & imaging parameters	N/A
Area of acquisition	N/A

Diffusion MRI ☐ Used ☒ Not used

Preprocessing

Preprocessing software	N/A
Normalization	N/A
Normalization template	N/A
Noise and artifact removal	N/A
Volume censoring	N/A

Statistical modeling & inference

Model type and settings	N/A
Effect(s) tested	N/A

Specify type of analysis: ☐ Whole brain ☐ ROI-based ☐ Both

Statistic type for inference (See Eklund et al. 2016)	N/A
Correction	N/A

Models & analysis

n/a	Involved in the study
☐	☐ Functional and/or effective connectivity
☐	☐ Graph analysis
☐	☐ Multivariate modeling or predictive analysis

Functional and/or effective connectivity	N/A
Graph analysis	N/A
Multivariate modeling and predictive analysis	N/A
