# Supplementary material for: ABCC9-related Intellectual disability Myopathy Syndrome is a KATP channelopathy with loss-of-function mutations in ABCC9
Source: Nat Commun. 2019 Oct 1;10:4457. doi: 10.1038/s41467-019-12428-7 (PMC6773855; doi:10.1038/s41467-019-12428-7)
Supplement: Supplementary file 4 — Description of Additional Supplementary Files [file 41467_2019_12428_MOESM4_ESM.pdf]

Description of Additional Supplementary Files

Supplementary Data 1:

Shared variants between patient 1-2 and 2-1 after WGS analysis and allele frequencies according to 1000 genomes (phase3) and ExAC (all and Finnish population).

Supplementary Movie 1:

High-speed video imaging of ventricular area of SUR2-STOP wildtype zebrafish at 5 dpf. Three entire cardiac cycles are shown which are slowed down by a factor of 5.

Supplementary Movie 2:

High-speed video imaging of ventricular area of SUR2-STOP mutant zebrafish at 5 dpf. Three entire cardiac cycles are shown which are slowed down by a factor of 5. Note reduced contractility compared to wildtype.
